# Brain activity during cognitive-motor tasks in older adults with cognitive frailty: a fNIRS study

**DOI:** 10.3389/fnagi.2025.1682050

**Published:** 2025-11-10

**Authors:** Yin-Hsiang Wang, Yea-Ru Yang, Nai-Chen Yeh, Pei-Yun Li, Ray-Yau Wang

**Affiliations:** Department of Physical Therapy and Assistive Technology, National Yang Ming Chiao Tung University, Taipei, Taiwan

**Keywords:** cognitive frailty, brain activity, cognitive-motor task, older adults, functional near-infrared spectroscopy

## Abstract

**Background:**

Cognitive frailty has recently drawn increasing attention in the context of elderly healthcare. While structural brain alterations in older adults with cognitive frailty have been previously explored, functional brain changes particularly during cognitive-motor tasks remain poorly understood. This study aimed to investigate functional brain activity during such tasks and its relationships with task performance to deepen understanding toward cognitive frailty.

**Methods:**

This cross-sectional study included cognitive frail and healthy older adults. Brain activity of bilateral prefrontal, supplementary motor area, and premotor cortex during two differently challenging cognitive-motor tasks and usual walking was measured by functional near-infrared spectroscopy. Cognitive-motor task performance, usual walking speed, physical performance, and executive function were also measured. Generalized estimating equation was used to analyze between tasks and populations. Pearson and Spearman’s correlation were used to examine relationships between task performance and brain activity.

**Results:**

Older adults with cognitive frailty activated all brain regions more during both cognitive-motor tasks than usual walking (*p* < 0.05) as healthy control. However, neither group showed increased brain activation during the difficult task compared to the easier (*p* > 0.05), despite significant task performance decline (*p* < 0.05). In cognitive frail older adults, activity of bilateral supplementary motor area and left premotor cortex correlated negatively with performance on the difficult task (*p* < 0.05). Reduced performance in executive and physical functions were also noted in older adults with cognitive frailty (*p* < 0.05).

**Conclusion:**

This study explored possible functional brain alterations of older adults with cognitive frailty, including neural reserve, capacity limitations, and neural inefficiency. The findings possibly contribute to identification of cognitive frailty, and intervention modulating such brain alterations is warranted in future studies.

## Introduction

The term “cognitive frailty” has been proposed to indicate the co-existence of physical frailty and mild cognitive impairment (MCI) (Sugimoto et al., 2022). Frailty is viewed as a transitional state between robustness and functional dependency (Fried et al., 2001), whereas MCI represents an intermediate cognitive stage between normal aging and early dementia (Petersen, 2004). The pooled prevalence of cognitive frailty is reported to range from 7 to 16% in community dwelling older adults (Qiu et al., 2022). Cognitive frailty not only predicts mortality and dementia more accurately than frailty alone (Bu et al., 2021) but also poses elevated risk of falls and disability to older adults, with odds ratios exceeding isolated physical frailty or MCI (Rivan et al., 2021). The increased risk, especially for falls, likely arises from the concurrence of cognitive and motor deficits compromising attentional control, responding to external environment, and internal motor planning for movement executions during daily activities (Yoshiura et al., 2022; Liu-Ambrose et al., 2008; Soto-Varela et al., 2020). Reviews have proposed that frailty and cognitive decline may share similar mechanisms, including chronic inflammation, impaired hypothalamic–pituitary axis stress responses, imbalanced energy metabolism, mitochondrial dysfunction, oxidative stress, and neuroendocrine dysfunction (Ma and Chan, 2020). These systemic changes may further affect the brain of older adults with cognitive frailty (Lauretani et al., 2020). Given the adverse health consequences associated with cognitive frailty, it is essential to further explore the phenotypic characteristics of individuals with cognitive frailty. Nonetheless, most existing studies have primarily focused on the performance characteristics of cognitive frailty, with limited evidence addressing brain alterations in individuals with this condition.

While previous studies have primarily focused on structural brain alterations in cognitive frailty, such as volumetric or diffusion abnormalities in hippocampal and subcortical regions (Yoshiura et al., 2022; Wan et al., 2020a; Wan et al., 2020b), little is known about *how functional brain responses of cognitive frailty adapt or fail* during cognitive-motor challenges. Understanding these *functional dynamics*, rather than static structural changes, may provide earlier and more sensitive indicators of cognitive frailty. Such functional brain changes have only been observed in older adults with frailty or MCI (Maruya et al., 2021; Cheng et al., 2021; Doi et al., 2013). The right prefrontal cortex (PFC) of prefrail older adults was found to activate less than that of robust ones during cognitive tasks, but show greater activation during walking compared to the resting condition, which was not observed in robust controls. This suggests a reduced neural capacity in prefrail older adults to meet cognitive task demands and a tendency toward brain overactivation during walking tasks (Maruya et al., 2021). Moreover, in a single group study, Cheng et al. noted that the PFC, premotor cortex (PMC), and supplementary motor area (SMA) of prefrail older adults did not show increased activation during cognitive dual-task walking compared to usual walking. They speculated that the aging brains of prefrail individuals might lack the capacity to sufficiently enhance brain activity to manage dual-task conditions (Cheng et al., 2021). As for older adults with MCI, Doi et al. (2013) conducted a single group study reporting that their PFC activated more during cognitive dual task walking than during usual walking, and the activation correlated with executive function. It seems that brain activation may vary across different populations and task conditions. Establishing characteristics of specific elder populations could possibly help with early identification and early intervention in senior health care, which is increasingly important in the aging society nowadays. As a cognitively and motorly deficient population, daily activities that normally involve both cognitive and motor challenges may already yield difficulties for people with cognitive frailty (Cadore et al., 2015). However, most previous research in frailty or mild cognitive impairment has examined only single-level dual-task paradigms. In contrast, our study employed a graded cognitive-motor paradigm using square-stepping exercises (SSE) to explore how increasing task demands modulate cortical activation. This approach allows for the identification of functional characteristics that may uniquely distinguish older adults with cognitive frailty.

Cognitive-motor tasking is essentially important in daily lives, and performance of it is related to falls of older adults (Muir-Hunter and Wittwer, 2016). Among, the SSE is a physical activity that involves stepping in specific patterns on a grid-like surface. The SSE patterns can range from simple to complex, depending on the direction and sequence of steps (Shigematsu and Okura, 2006). These patterns simultaneously challenge both physical and cognitive functions; therefore, SSE is commonly employed to enhance balance, coordination, and cognitive performance, particularly in older adults (Wang et al., 2021). This study examined brain activity in response to various cognitive-motor challenges induced by SSE in older adults with cognitive frailty, compared to healthy older adults, to provide insight into how cognitive frailty affects brain activation during cognitive-motor tasks.

## Methods

### Study design

**Figure 1:**
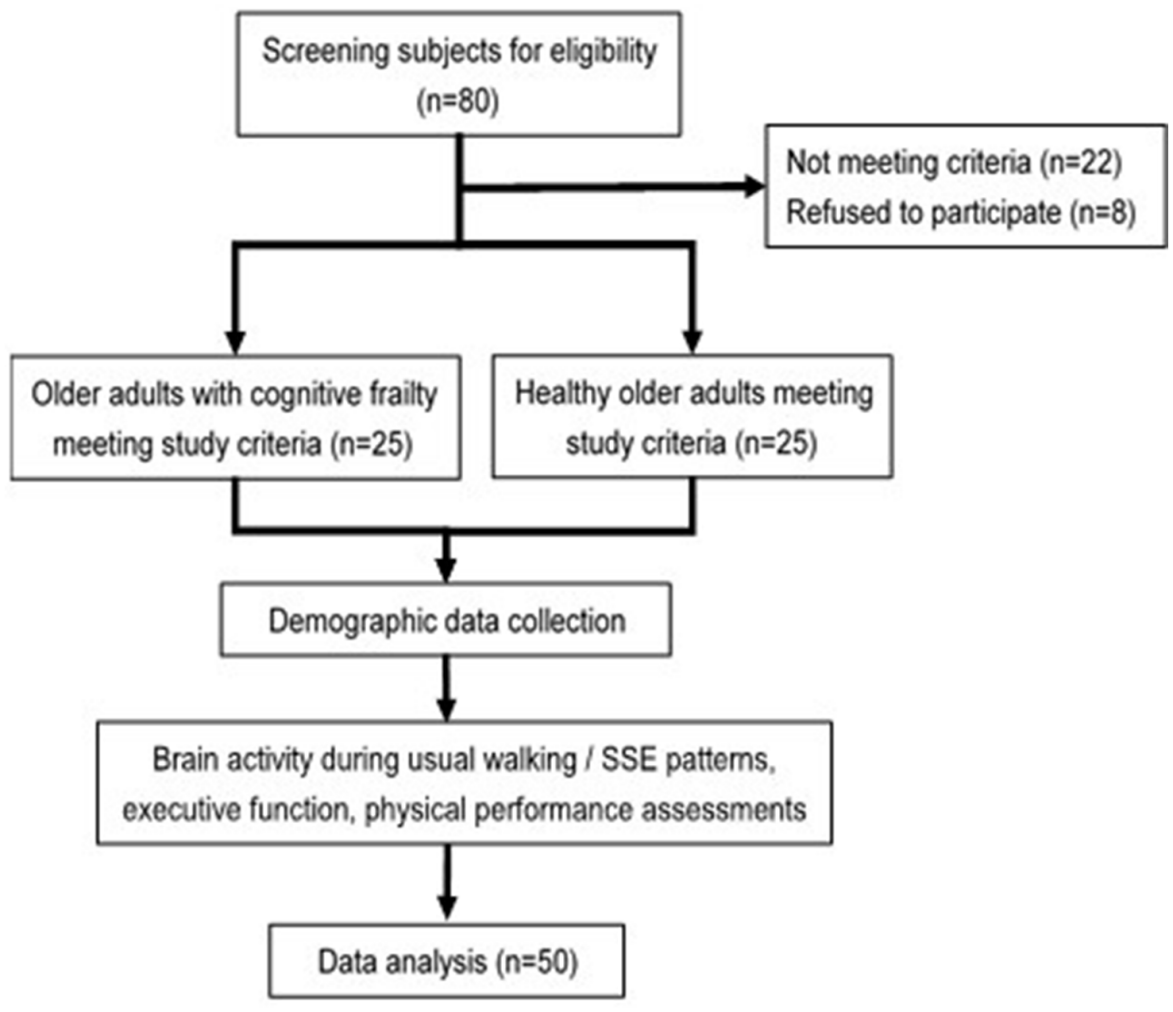
Flow chart of the study.

This was a cross-sectional study, study flowchart shown as Figure 1. Study protocol was approved by the Institutional Review Board of National Yang Ming Chiao Tung University (YM110152F, NYCU112078AE) and preregistered on the ClinicalTrials.gov website^1^ (NCT05891574, NCT05173363). All participants were provided with and signed the written informed consent form of the study. Age, gender, education level, frailty status, and global cognition status (MMSE and MoCA scores) were collected as demographic characteristics. As for assessment, brain activity during SSEs and usual walking, SSEs performance, walking speed, executive function, and physical performance were measured.

### Participants

Participants were recruited from the surrounding communities and senior health care centers. The inclusion criteria of older adults with cognitive frailty included: (1) age ≧ 65 years old, (2) the presence of at least 1 characteristic of the 5 following physical characteristics for frailty as defined by Chen et al. (2014): unintentional body weight loss, exhaustion, weakness, slow gait speed, and low physical activity level, (3) with MCI as defined by mini-mental state examination (MMSE) score ≧ 24 and Montreal Cognitive Assessment (MoCA) score <26 (Petersen, 2004; Nasreddine et al., 2005), and (4) ability to walk independently for 1 min without assistive devices. The inclusion criteria of healthy older adults included: (1) age ≧ 65 years old, (2) the absence of frailty characteristics (Chen et al., 2014), (3) mini-mental state examination (MMSE) score ≧ 24 and Montreal Cognitive Assessment (MoCA) score ≧ 26, and (4) ability to walk independently for 1 min without assistive devices. The exclusion criteria for both populations were: (1) central nervous system disorders (such as dementia, stroke, Parkinson’s disease, spinal cord injury), and (2) any unstable physical condition, psychiatric disorder, and other neurological disorder or diagnosed with learning disability which may affect participating the study.

### Measurements

#### Brain activity

**Figure 2:**
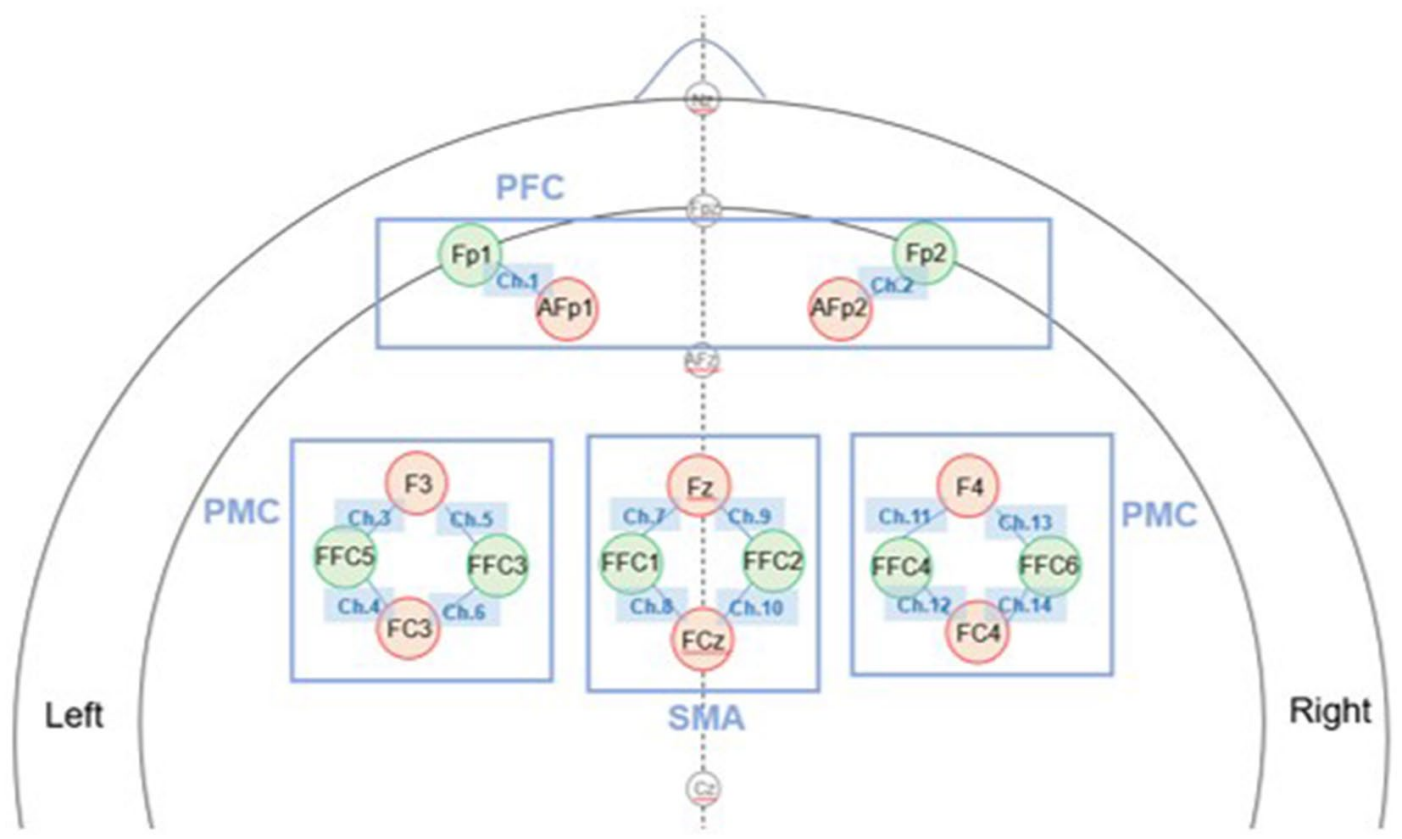
Configuration of fNIRS optodes and measurement channels. Optode placement was determined according to the international 10–5 system (top view). The setup consisted of 16 optodes (8 sources in red and 8 detectors in green) forming 14 measurement channels covering the bilateral prefrontal cortex (PFC), premotor cortex (PMC), and supplementary motor area (SMA). Each line connecting a source and a detector represents one measurement channel.

Brain oxygenation indicating the brain activity during tasking was measured by a wearable functional near-infrared spectroscopy system (fNIRS) (NIRSport2, NIRx Medical Technologies LLC) (Funahashi and Andreau, 2013). The fNIRS system consisted of 16 optodes (8 light sources and 8 detectors) arranged according to the international 10–5 system, forming 14 measurement channels covering the bilateral PFC, PMC, and SMA (Figure 2). The bilateral PFC, PMC, and SMA were the regions of interest measured in this study, since PFC is involved in executive function (Friedman and Robbins, 2022), and PMC and SMA are involved in balance and motor function (Wittenberg et al., 2017). To monitor the concentration of oxyhemoglobin (HbO) in local brain areas, near infrared light was produced by the sources and collected by the detectors, with a sampling rate of 7.81 Hz. The optodes were attached to specific locations based on the international 10–5 system (Oostenveld and Praamstra, 2001). For data processing, several steps were performed to ensure signal quality. First, data rejection was performed based on the coefficient of variation (CV). Channels with CVchan ≥ 15% or trials with CVtrial ≥ 10% were excluded (Lu et al., 2015). On average, the channel rejection rate was 2.0% per participant, and the trial rejection rate was 0.24% per channel. Next, data processing was conducted using the HOMER2 package. Raw intensity signals were converted to optical density, followed by filterings, which were used to correct signal bias. Wavelet filtering was first applied to remove motion artifacts, identified as the time points when signal changes exceeded 10 times the standard deviation within each channel (Huang and Li, 2024). Next on, bandpass filtering (low-cutoff frequency at 0.01 Hz, high-cutoff frequency at 0.2 Hz) (Hocke et al., 2018) was used to eliminate the influences of heartbeat, respiration, and low frequency signal drifts. These preprocessed signals were then computed into oxyhemoglobin concentration changes by the modified Beer–Lambert law.

#### Task paradigm

Brain activity was measured during three task conditions: usual walking, square-stepping exercise pattern 1 (SSE1), and square-stepping exercise pattern 2 (SSE2). Each task was performed twice, yielding a total of six trials, with a 1-min standing rest between trials. The tasks were presented in a semi-randomized alternating order that was pre-generated prior to data collection and applied consistently across participants: *usual walking → SSE1 → SSE2 → SSE1 → SSE2 → usual walking*. This design was intended to minimize participants’ anticipation of the upcoming task while maintaining protocol consistency. A fully randomized or counterbalanced design was not adopted because the number of possible task combinations (up to 30 even after excluding consecutive repetitions of the same task) was impractical for the present sample size. Similar approaches using fixed or pre-defined walking task sequences have been reported in previous fNIRS studies examining motor and cognitive-motor tasks (Doi et al., 2013; Maidan et al., 2016). Each task of this study was described in the following:

(1) Usual walking: participants walked continuously in a loop pattern along a 25-m pathway without stopping at the ends. Although turning might cause minor deceleration, this effect was consistent across participants.
(2) SSE1: participants performed SSE pattern 1 on the SSE mat at their comfortable speed, and repeat the pattern for 1 min.
(3) SSE2: participants performed SSE pattern 2 on the SSE mat at their comfortable speed, and repeat the pattern for 1 min.

**Figure 3:**
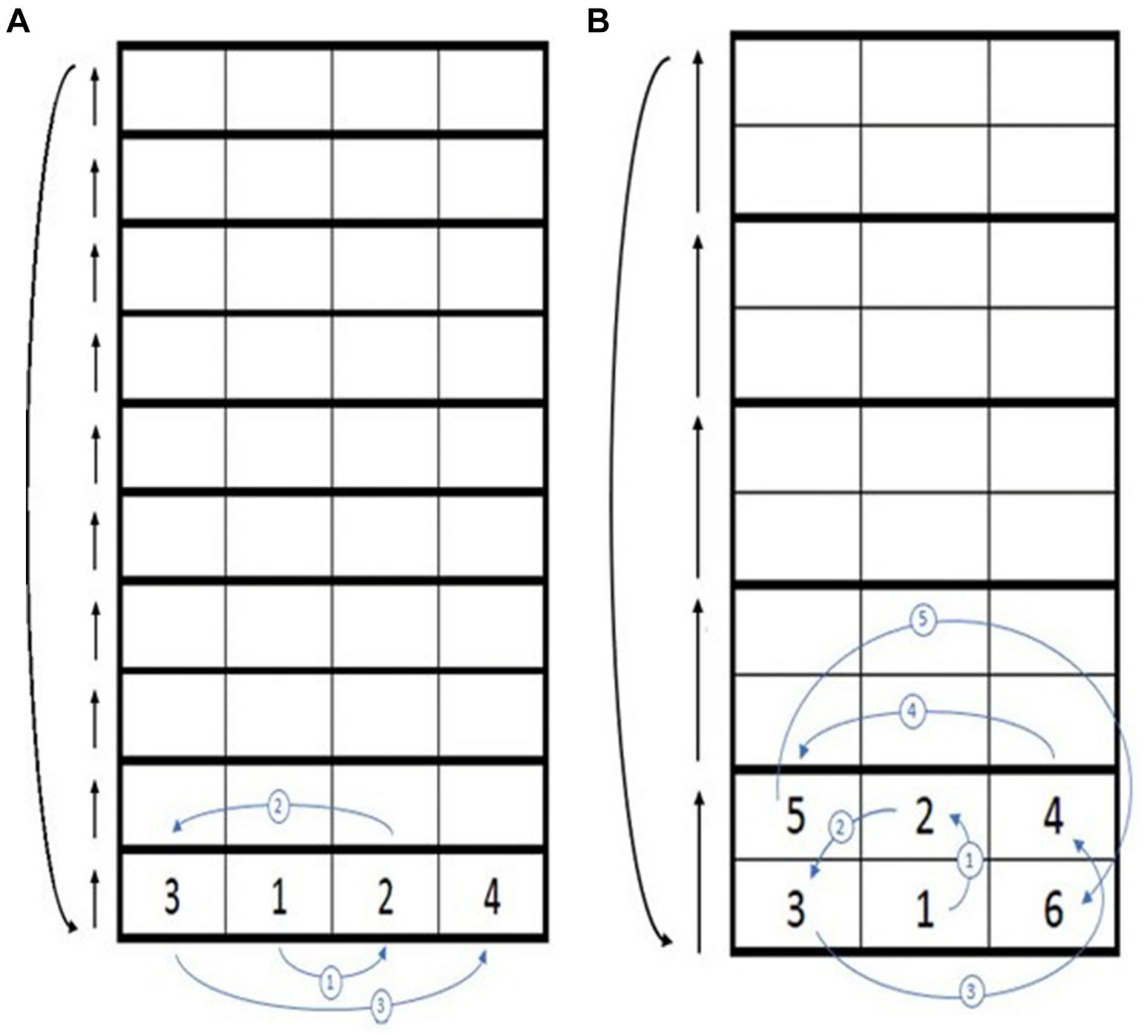
Square-stepping exercise (SSE) patterns with different difficulty levels. **(A)** SSE pattern 1; **(B)** SSE pattern 2. The numbers in the squares (1, 2, 3, …) indicate the stepping sequence, beginning with the left foot and alternating between feet according to the numbered order. In SSE1, a four-step cycle is repeated, whereas SSE2 consists of a six-step cycle involving forward, oblique, and backward movements, representing a higher level of motor and cognitive complexity. After completing one full sequence, participants advance forward by four squares in SSE1 or six squares in SSE2 to begin the next cycle, maintaining a continuous stepping flow throughout the task.

The SSE 1 and SSE 2 patterns in the present study were adopted from elementary2-pattern2 and intermediate3-pattern20 from the SSE instructor guiding handbook, respectively. Motor-wise, SSE1 consisted primarily of forward and lateral steps with shorter step distances, while SSE2 required forward, oblique, and backward steps with longer stepping distances, resulting in a longer single-leg stance period and greater postural control demand. Cognitive-wise, SSE1 involved memorizing and executing a four-step sequence, whereas SSE2 extended the sequence to six steps, imposing higher working-memory and information processing demands. Figure 3 illustrates the two SSE patterns.

#### Cognitive-motor task performance (SSE performance)

The number of the correct steps that participants completed in 1 min during SSEs were recorded (correct step was defined as stepping in the squares without stepping on the lines). The average of the two trials for each pattern was used to indicate the SSE performance. The higher the number, the better the SSE performance.

#### Walking speed

The distance walked during 1-min usual walking condition was recorded to calculate for the walking speed. The average of the two trials was used for analysis.

#### Executive function

##### Working memory

The Digit Span Test (DST), a subtest of the Wechsler Adult Intelligence Scale-Revised, was used to assess working memory. It is a commonly used measure with a test–retest reliability of 0.83 (Groth-Marnat and Baker, 2003). The test consists of a forward and backward span. The sequence ranges from 3 to 9 digits for the forward span and 2 to 8 digits for the backward span. In this study, the total score was calculated as the sum of both spans.

##### Cognitive flexibility

Trail Making Test part B (TMT-B) is used to indicate the executive function especially in task-shifting (cognitive flexibility). The modified Chinese version TMT-B containing 12 numbers (1–12) and 12 Chinese zodiac signs was used in this study (test–retest reliability *r* = 0.89) (Wang et al., 2018). Participants drew lines to connect numbers in ascending order but with the additional task of alternating between numbers and Chinese zodiac signs (i.e., 1-rat-2-ox-3-tiger, etc.). The time needed to complete the test was recorded.

##### Inhibitory control

The Stroop Color and Word Test (SCWT)-Incongruent was used in this study to assess executive function, particularly selective attention (inhibitory control), with a high test–retest reliability of 0.91 (Wang et al., 2018). During the test, the participants were asked to name the color of the ink in which incongruent color word was printed. The test was scored with the number of correct answers within 45 s.

#### Physical performance

##### Functional mobility

The timed up and go test (TUG) (ICC = 0.98) (Steffen et al., 2002) was used to evaluate functional mobility in this study. During the test, the participants were instructed to stand up from a chair, walk as fast as possible for 3 meters, turn, walk back to the chair, and sit down. The time needed to complete the test was recorded. The test was repeated twice, with a 30-s rest in between, and the average was used for data analysis.

##### Balance

The Berg-balance scale (BBS) was used to measure the balance performance in this study. The BBS is a 14-item measurement of balance with test and retest reliability of 0.886 (Viveiro et al., 2019). Each item is scored on a 5-point scale, ranging from 0 to 4. Total score of BBS ranges from 0 to 56.

##### Lower extremity muscle strength

The five times sit to stand test (FTSTS) was used to indicate the lower extremity strength in this study. The FTSTS has a good test–retest reliability of 0.89 (Tiedemann et al., 2008). Participant stood up and sat down using a standard chair, with arms folding across chest, as quickly as possible for five times. The time needed to complete the test was recorded. The test was done twice with a 30-s rest in between, and the average was used for data analysis.

### Sample size calculation

The required sample size was estimated using G*Power 3.1, with a type I error of 0.05 and a statistical power of 80%. As no prior studies were available for direct reference, a medium effect size (*f* = 0.25) and a medium correlation coefficient (*r* = 0.4) were adopted for the calculation (Viveiro et al., 2019). The minimum total sample size was estimated to be 20 based on the within–between interaction model and 46 based on the correlation model. To ensure adequate power, the larger estimate (*n* = 46) was selected. Considering a potential 5–10% attrition rate during data processing, the target sample size was set at 50 participants (25 per group). The final sample size thus exceeded twice the number required for the within–between interaction analysis, supporting sufficient power to detect subtle group and task-related differences. However, this sample size was powered to detect medium effect sizes and may have been insufficient to identify small or non-significant effects, which should be interpreted with caution.

### Statistical analysis

SPSS version 25.0 (SPSS Inc., Chicago, IL, United States) was used to analyze the data in this study. Shapiro–Wilk test was used to confirm for normal distribution in each population. Demographic characteristics between populations were compared by independent *t*-test (for comparison of age), Mann–Whitney *U* test (for educational level, frailty status, MoCA, MMSE), or Chi square test (for gender). In this study, generalized estimating equations (GEE) were employed to model population-averaged effects across groups and task conditions. This approach was chosen because it accounts for within-subject correlations arising from repeated measures and was robust to violations of normality assumptions. Given that our dataset included both continuous and ordinal variables, GEE provided a more flexible and reliable framework for estimating group and condition effects (Hubbard et al., 2010). Therefore, the brain activity in different task conditions and different populations (groups) was compared by GEE, with age and education level as the adjusted covariates since significant group differences were observed in these variables. The multiple comparisons were then adjusted by Bonferroni corrections. The SSE, executive function, and physical performance were also analyzed by GEE for group comparisons, with age and education level as covariates since significant group differences were observed in these variables. The significant level was set at *p* ≤ 0.05. For correlation analyses, Pearson’s correlation was used when both variables were normally distributed, and Spearman’s rank correlation was used when one or both variables violated normality assumptions, to examine the relationships between SSEs performance and brain activity. *p* value of the multiple correlation analysis was adjusted to 0.05/12 in each population.

## Results

Figure 1 illustrates the flow chart of the present study. Twenty-five cognitive frailty participants and 25 healthy control were recruited. Demographic characteristics of both populations are listed in Table 1. There were 20 females and 5 males in cognitive frailty group and 22 females and 3 males in the healthy control group. A mean of 1.5 frailty characteristics was shown in cognitive frailty group while 3 among the 25 participants were identified as frail (Fried score = 3, *n* = 3), and the other 22 participants were identified as prefrail (Fried score = 1, *n* = 15; Fried score = 2, *n* = 7). The mostly matched frailty criterion was weakness (*n* = 9). Low physical activity level and exhaustion were each presented in 8 participants, while slowness and weight loss criteria were, respectively, noted in 7 and 6 participants, respectively. The frailty status and cognitive function of the two groups revealed significant difference, corresponding to their population features. An average score of 22.8 on MoCA and 26.3 on MMSE were noted in cognitive frailty group, and 27.6 on MoCA and 28.5 on MMSE in healthy control group. The group differences were also presented in age and education level (*p* < 0.001; *p* < 0.001). The cognitive frailty group (mean age: 78.4 years; mean education level: 10.4 years) had a higher mean age and a lower mean education level compared to the healthy control group (mean age: 70.9 years; mean education level: 14.4 years). Therefore, age and education level were used as covariates in the comparison analysis between the populations.

**Table 1:** Demographic characteristics of participants (*n* = 50).

Group	Cognitive frailty (*n* = 25)	Healthy control (*n* = 25)	*P*-value
Gender (female/male)^c^	20/5	22/3	0.440
Age (years)^a^	78.4 ± 5.9	70.9 ± 2.7	<0.001
Education (years)^b^	10.4 ± 3.3	14.4 ± 2.6	<0.001
Frailty status^b^	1.5 ± 0.7	0 ± 0	<0.001
MoCA^b^	22.8 ± 2.4	27.6 ± 1.6	<0.001
MMSE^b^	26.3 ± 1.5	28.5 ± 1.2	<0.001

Data presented as numbers or mean ±standard deviation. MoCA, Montreal Cognitive Assessment; MMSE, Mini-mental state examination.

^a^*t*-test.

^b^Mann–Whitney *U* test.

^c^Chi square test.

### Brain activities and performance during different cognitive-motor tasks

**Figure 4:**
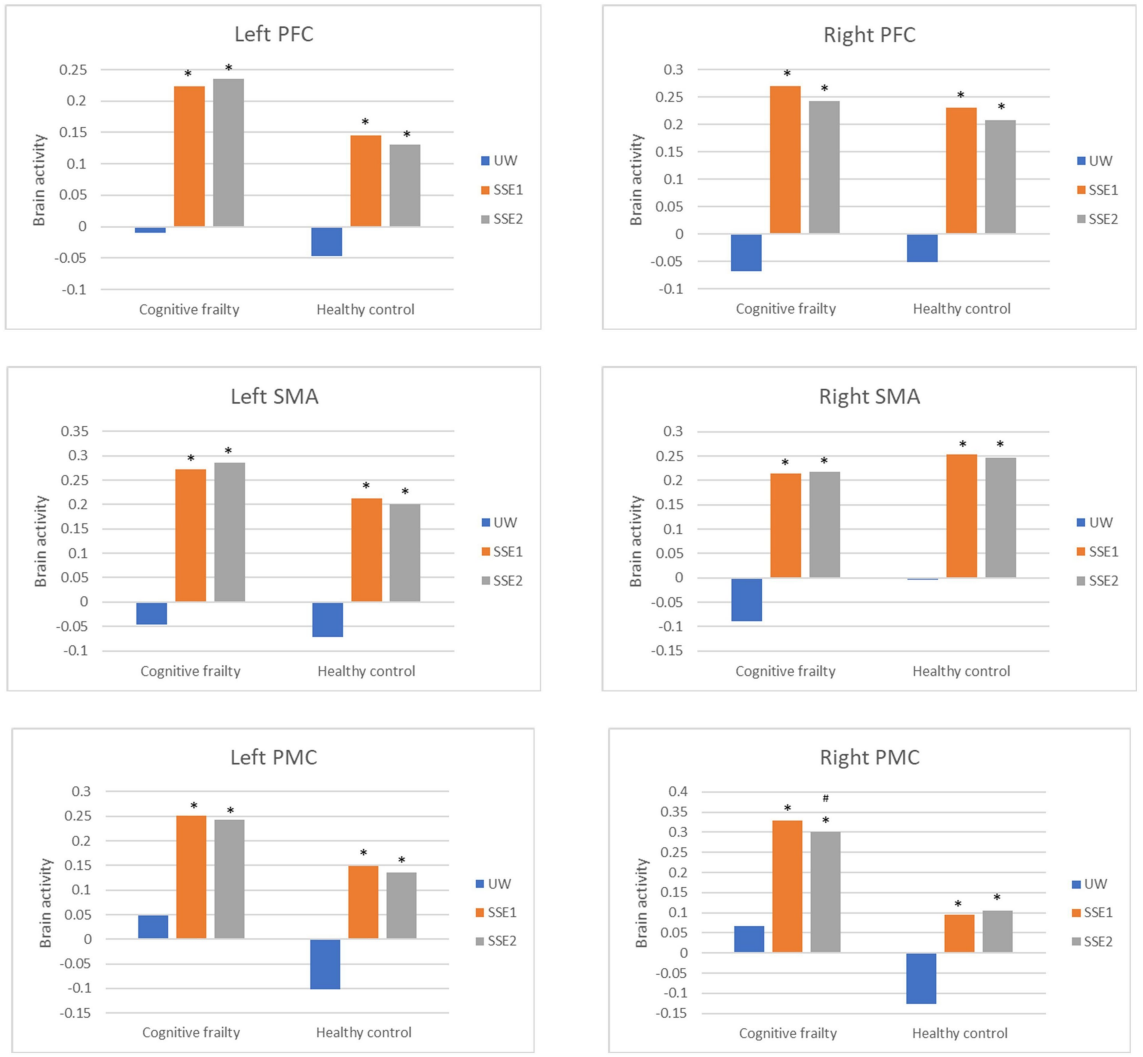
Brain activation between populations (cognitive frailty vs. healthy controls) and conditions (UW, SSE1, SSE2). Bars represent brain activities adjusted for age and education level. SSE, square-stepping exercise; UW, usual walking; PFC, prefrontal cortex; SMA, supplementary motor area; PMC, premotor cortex. *p < 0.05 compared to UW (after Bonferroni correction). #*p* < 0.05 compared to SSE1 (after Bonferroni correction).

Brain activities were compared between populations (cognitive frailty/healthy) and conditions (UW/SSE1/SSE2). As shown in Table 2 and Figure 4, after adjustments of age and education level, all of the measured brain regions (left and right PFC, SMA, and PMC) revealed significant condition effect (*p* < 0.05). In addition, the right PMC also showed significant population effect (Wald *χ*^2^ = 6.068, *p* < 0.05) and population by condition interaction (Wald *χ*^2^ = 11.113, *p* < 0.05). In the results of multiple comparisons after Bonferroni correction, both SSE1 and SSE2 induced higher brain activity in all measured brain regions than usual walking in both older adults with cognitive frailty and healthy older adults (*p* < 0.05). However, there was no significant difference in bilateral PFC, bilateral SMA, and left PMC activity when performing these two different SSE patterns in both populations, as well as in the right PMC of healthy older adults (*p* > 0.05). In the right PMC of older adults with cognitive frailty, SSE2 induced less brain activity than SSE1 did (*p* < 0.05). Significant group difference was not revealed in all measured brain areas (*p* > 0.05).

**Table 2:** Brain activities between populations (cognitive frailty/healthy) and conditions (UW/SSE1/SSE2).

Brain regions	Population	UW	SSE1	SSE2	Population effect		Condition effect		Population*condition	
		Estimated mean (standard error)	Estimated mean (standard error)	Estimated mean (standard error)	Wald *χ*^2^	*P*	Wald *χ*^2^	*P*	Wald *χ*^2^	*P*
Left PFC	Cognitive frailty	−0.010 (0.050)	0.224 (0.050) *	0.235 (0.050) *	0.966	0.326	40.976	<0.001	1.370	0.504
	Healthy control	−0.047 (0.053)	0.146 (0.053) *	0.131 (0.053) *						
Right PFC	Cognitive frailty	−0.067 (0.048)	0.270 (0.048) *	0.243 (0.048) *	0.076	0.783	83.022	<0.001	0.655	0.721
	Healthy control	−0.051 (0.051)	0.231 (0.051) *	0.208 (0.051) *						
Left SMA	Cognitive frailty	−0.047 (0.047)	0.272 (0.047) *	0.286 (0.047) *	0.768	0.381	77.958	<0.001	1.294	0.524
	Healthy control	−0.072 (0.046)	0.212 (0.046) *	0.200 (0.046) *						
Right SMA	Cognitive frailty	−0.090 (0.052)	0.214 (0.052) *	0.217 (0.052) *	0.453	0.501	242.207	<0.001	0.876	0.645
	Healthy control	−0.004 (0.051)	0.253 (0.051) *	0.246 (0.051) *						
Left PMC	Cognitive frailty	0.048 (0.049)	0.251 (0.049) *	0.242 (0.049) *	2.937	0.087	43.645	<0.001	0.518	0.772
	Healthy control	−0.102 (0.048)	0.149 (0.048) *	0.136 (0.048) *						
Right PMC	Cognitive frailty	0.066 (0.059)	0.328 (0.059) *	0.301 (0.059) *^,#^	6.068	0.014	34.147	<0.001	11.113	0.004
	Healthy control	−0.126 (0.058)	0.095 (0.058) *	0.105 (0.058) *						

Data presented as estimated mean (standard error) after adjusted by age and education level. Abbreviations: SSE, square-stepping exercise; UW, usual walking; PFC, prefrontal cortex; SMA, supplementary motor area; PMC, premotor cortex.

*p < 0.05 compared to UW (after Bonferroni correction).

^#^*p* < 0.05 compared to SSE1 (after Bonferroni correction).

Cognitive-motor task performance was compared between populations (cognitive frailty/healthy) and conditions (SSE1/SSE2). As shown in Table 3, after adjustments of age and education level, the GEE model revealed significant population effect (*p* < 0.05) and condition effect (*p* < 0.001), but not population by condition interaction. In the multiple comparisons, both population groups completed fewer correct steps during SSE2 than SSE1. Older adults with cognitive frailty completed comparable steps as healthy older adults during SSE1 (*p* > 0.05), but completed less correct steps than healthy older adults during SSE2 (*p* < 0.001).

**Table 3:** Group comparisons in SSE/physical/cognitive performance.

Measures	Cognitive frailty	Healthy control	Population effect		Condition effect		Population*condition	
	Estimated mean (standard error)	Estimated mean (standard error)	Wald *χ*^2^	*P*	Wald *χ*^2^	*P*	Wald *χ*^2^	*P*
UW (m/s)	0.741 (0.002)*	1.016 (0.002)	5923.004	<0.001	–	–	–	–
SSE1 performance (steps/min)	101.653 (5.677)	116.127 (5.677)	4.920	0.027	237.232	<0.001	1.426	0.232
SSE2 performance (steps/min)	43.133 (5.677)*^,#^	66.027 (5.677)^#^						
DST (numbers)	16.084 (0.286)*	18.156 (0.286)	13.090	<0.001	–	–	–	–
TMT-B (s)	117.490 (3.450)*	97.774 (3.450)	8.162	0.004	–	–	–	–
SCWT – incon	36.877 (0.709)	39.603 (0.709)	3.699	0.054	–	–	–	–
TUG (s)	12.730 (0.792)*	8.298 (0.792)	7.821	0.005	–	–	–	–
BBS (points)	52.970 (0.407)	51.990 (0.407)	1.446	0.229	–	–	–	–
FTSTS (s)	9.772 (0.049)*	8.776 (0.049)	102.125	<0.001	–	–	–	–

Data presented as estimated mean (standard error) after adjusted by age and education level. UW, usual walking; SSE, square-stepping exercise; MoCA, Montreal Cognitive Assessment; DST, digit span test; TMT-B, trail making test part B; SCWT_incon, Stroop test-incongruent part; TUG, Timed up and go test; FTSTS, Five time sit to stand test.

*p < 0.05 compared to healthy control.

^#^*p* < 0.05 compared to SSE1.

### Population comparisons in executive function and physical performance

Table 3 reports the executive function and physical performance of both populations. All group comparisons were adjusted by age and education level. Older adults with cognitive frailty demonstrated slower walking speed (*p* < 0.001), TUG (*p* = 0.05), and FTSTS (*p* < 0.001) than healthy older adults, whereas no significant group difference was found in BBS (*p* = 0.229). Regarding executive functions, older adults with cognitive frailty demonstrated worse performance in DST (*p* < 0.001) and TMT-B (*p* = 0.004) in comparison with healthy older adults. The result of SCWT-incongruent test, however, did not reveal significant group difference (*p* = 0.054).

### Relationships between cognitive-motor task performance and brain activity

**Figure 5:**
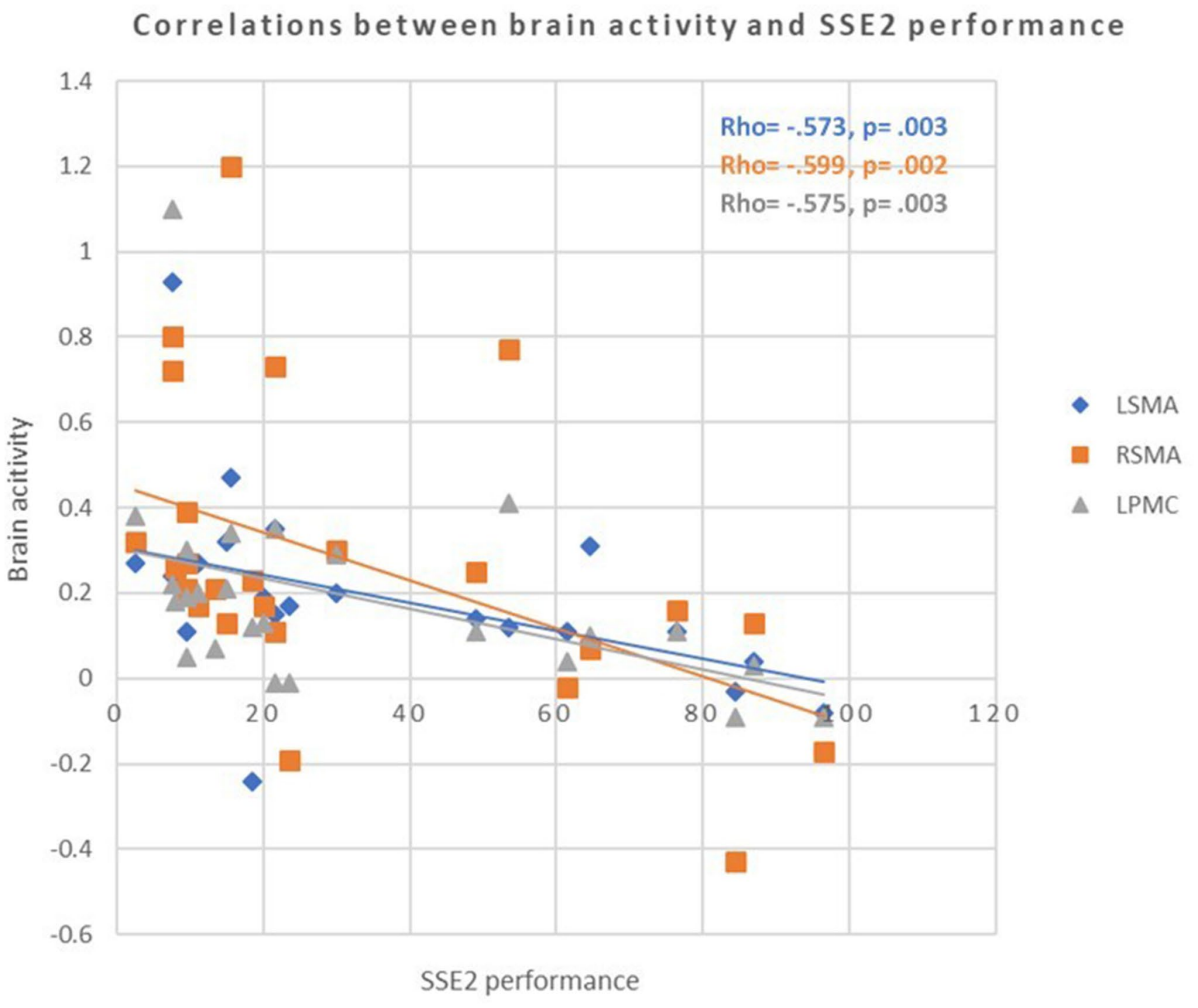
Scatter plots showing the correlations between brain activity and square-stepping exercise (SSE2) performance in older adults with cognitive frailty. Greater activation in the left and right supplementary motor areas (LSMA, RSMA) and left premotor cortex (LPMC) was associated with poorer performance, indicating neural inefficiency. Solid lines represent linear regression fits (*p* < 0.0042, Bonferroni-corrected).

The relationships between SSEs performance and brain activity are reported in Table 4 and Figure 5. Significant correlations were only found in cognitive frailty group. When performing SSE 2, the activity of left and right SMA (rho = −0.573, *p* = 0.003; rho = −0.599, *p* = 0.002), as well as left PMC (rho = −0.575, *p* = 0.003) in older adults with cognitive frailty was negatively correlated to the performance of SSE 2.

**Table 4:** Correlations between brain activity and task performance.

Brain activity	Cognitive frailty				Healthy control			
	SSE1 performance		SSE2 performance		SSE1 performance		SSE2 performance	
	*R*/rho	*p*-value	*R*/rho	*p*-value	*R*/rho	*p*-value	*R*/rho	*p*-value
Left PFC	0.175^b^	0.404	−0.126^b^	0.558	0.170^a^	0.448	−0.235^b^	0.291
Right PFC	−0.003^a^	0.988	−0.100^b^	0.642	0.029^a^	0.900	−0.261^a^	0.241
Left SMA	−0.275^a^	0.183	−0.573^b^	0.003*	0.069^a^	0.742	−0.035^a^	0.869
Right SMA	0.033^a^	0.877	−0.599^b^	0.002*	0.168^a^	0.423	−0.084^a^	0.691
Left PMC	−0.243^a^	0.242	−0.575^b^	0.003*	0.150^a^	0.475	−0.172^a^	0.410
Right PMC	0.219^a^	0.293	−0.209^b^	0.327	0.365^a^	0.073	−0.100^a^	0.635

SSE, square-stepping exercise; PFC, prefrontal cortex; SMA, supplementary motor area; PMC, premotor cortex.

^a^By Pearson correlation.

^b^By Spearman’s correlation.

^*^p ≤ 0.0042.

## Discussion

This was the first study to demonstrate brain activity along with task performance during cognitive-motor tasks in older adults with cognitive frailty. Similar to healthy older adults, they showed greater activation in motor- and cognitive-related areas (bilateral PFC, SMA, PMC) during cognitive-motor tasks, such as SSE 1 and SSE 2, compared to usual walking. However, the increasing challenging levels of cognitive-motor tasks did not seem to further enhance brain activity in older adults with cognitive frailty or in healthy older population. The right PMC of older adults with cognitive frailty even showed less activity during the more difficult cognitive-motor task than the easier one. Regarding cognitive-motor task performance, both groups performed worse on the more difficult task compared to the easier one. Older adults with cognitive frailty performed comparably as healthy older adults on the easier task, but showed a significant decline on the more difficult task. It is also noteworthy that reduced activity in the right and left SMA and the left PMC during the higher cognitive-motor demanding task was associated with better task performance in older adults with cognitive frailty. In this population, reduced performance in working memory, cognitive flexibility, usual walking speed, functional mobility, and lower extremity muscle strength was significant, compared to healthy older adults.

In the present study, older adults with cognitive frailty demonstrated comparable performance in SSE1 to healthy older adults, with enhanced PFC, SMA, PMC activation to similar level. Previous studies have also reported increased brain activity in the PFC, SMA, and PMC during dual-task walking compared to usual walking in older adults (Bishnoi et al., 2021; Stuart et al., 2019). Additionally, in older adults with MCI, increased activity was observed specifically in the PFC (Doi et al., 2013). These findings suggest that cognitive-motor tasks rely heavily on the activity of these brain regions. It is known that PFC plays a major role in cognitive control including executive processing (Friedman and Robbins, 2022). The SMA and PMC are thought to be responsible for motor planning and execution (Wittenberg et al., 2017), and both are later said to help with linking cognition to actions. The SMA is reported to be related to cognitive flexibility and selective attention (Nachev et al., 2008), while PMC contributes to space perception before movements (Rizzolatti et al., 2002). The performance of SSE1 in the present study requires abilities such as executive function, balance, and lower extremity control, and PFC, SMA, and PMC are functionally related to such task demands. Therefore, although older adults with cognitive frailty exhibited greater vulnerability in multiple cognitive and physical functions compared to healthy older adults (Tables 1, 3), they still retained a certain degree of neural reserve, as demonstrated in the relatively simple cognitive-motor task.

However, as the cognitive-motor challenge increased, as required by SSE2 in the present study, both older adults with cognitive frailty and healthy older adults failed to enhance brain activation and exhibited decreased SSE2 performance compared to SSE1. This observation may indicate limitation in neural capacity (Callicott et al., 1999; Watanabe and Funahashi, 2014). In other words, the brain’s ability to enhance activation in response to increased task load has an upper limit. As a result, it cannot further activate to meet the demands of a more difficult task, leading to decreased performance. The neural capacity limitation observed in healthy older adults in the present study was also noted in a previous study (Steffener et al., 2020). Steffener et al. (2020) demonstrated that older adults exhibited no increase in brain activity during a complex cognitive task compared to a simpler task, as measured by whole-brain functional magnetic resonance imaging (fMRI). In the present study, we further identified this neural capacity limitation in older adults with cognitive frailty.

It also drew our attention that right PMC of older adults with cognitive frailty even showed less activity during the more difficult cognitive-motor task than the easier one. This aligns with a theoretical framework suggesting that lower-functioning older adults tend to exhibit decreased activation earlier than higher-functioning older adults when task demands exceed their neural resources, ultimately leading to decline in task performance (Clark et al., 2019; Kang et al., 2021; Reuter-Lorenz and Cappell, 2008). Although this theory has been largely discussed in relation to the PFC (Clark et al., 2019; Kang et al., 2021) rather than the PMC, we believe that older adults with cognitive frailty, as a lower-functioning population in the present study, likely experienced decreased activation of the right PMC due to the excessive task demands in SSE2. The right hemisphere is often considered dominant for visuospatial and attentional processing (Gunturkun et al., 2020). Specifically, the right PMC is part of a network linked with the right inferior parietal lobule, playing a crucial role in the perception of spatial relationships (Schintu et al., 2014). The right dorsal PMC is functionally connected to the prefrontal cortex, which governs high-level cognitive processes. As such, SSE2 performance may rely heavily on the right PMC, which exhibited early signs of decreased activation in older adults with cognitive frailty.

Given that older adults with cognitive frailty performed worse than healthy controls during SSE2 despite showing comparable cortical activation, we speculate that older adults with cognitive frailty may manage their brain resources less efficiently, that is, reduced neural efficiency (Lehmann et al., 2022). Indeed, the negative correlations between brain activity in the left SMA, right SMA, left PMC, and SSE2 performance in older adults with cognitive frailty further support this idea, indicating inefficient resource utilization. Another possible explanation for this negative relationship is compensatory overactivation, as reported in previous aging studies (Clark et al., 2019; Chatterjee et al., 2020), where lower-functioning individuals tend to overrecruit neural resources under equivalent task demands. Such over-recruitment may initially reflect an attempt to maintain performance but ultimately signal limited processing efficiency, aligning with the concept of neural inefficiency. Additionally, fatigue or attentional decline induced by the more demanding task may have contributed to increased activation accompanied by performance deterioration.

Our study not only demonstrated the possible inefficiency in older adults with cognitive frailty, but also the capacity limitations in both cognitively frail and healthy older adults. Neural efficiency is believed to be associated with human intelligence and cognitive reserve in the aging brain (Neubauer and Fink, 2009), whereas capacity limitation may reflect the brain’s limitation to recruit compensatory neural resources to meet task demands (Steffener et al., 2020). Investigating neural inefficiency and capacity limitation contributes to a better understanding of the brain mechanisms underlying task performance. The hemodynamic changes observed in individuals with cognitive frailty in present study provide insight into cognitive-frailty-related functional brain deviations, as they diverge from the typical aging trajectory. Identifying these functional brain alterations, reflecting neural reserve, capacity limitation, and inefficiency, may facilitate the early detection of cognitive frailty and help target older adults at risk before progression to more severe neuropathological conditions. Furthermore, longitudinal studies that continuously monitor neural changes in individuals with cognitive frailty are warranted to delineate the trajectory of disease development and prognosis. The importance of intervention is also underscored, as the aging brain remains plastic and responsive to training (Aron et al., 2022; Park and Bischof, 2013). Interventions designed to enhance neural efficiency and capacity may therefore help attenuate the neurodegenerative processes underlying cognitive frailty and reduce the risk of progression to dementia.

In addition to brain activity in different cognitive-motor tasks, this study also provided the physical and cognitive performance in older adults with cognitive frailty as compared with healthy older adults. Older adults with cognitive frailty demonstrated worse working memory, cognitive flexibility, functional mobility, and lower extremity strength as compared with healthy older adults, which were also noted in previous study (Yoshiura et al., 2022). However, the present study further documented alterations of specific executive domains, working memory and cognitive flexibility, in cognitive frailty. The identification of such insufficiency in these specific executive domains is important. The decreased working memory was found to relate to older adults’ judgment in motor planning, which may increase the fall risks (Liu-Ambrose et al., 2008). Worse cognitive flexibility may predict impaired physical function and mortality in older people (Vazzana et al., 2010). Therefore, interventions targeting these certain aspects of executive function should be further valued in the cognitive frailty population. Aside from the decreased grip strength found in the previous study (Yoshiura et al., 2022), our study reported reduced overall lower extremity strength, as well as impaired functional mobility and slower walking speed, in older adults with cognitive frailty. This deficiency in motor abilities have been shown essential for older adults’ major health issues (Soto-Varela et al., 2020; Cho et al., 2012; Rosmaninho et al., 2021). Taken together, the executive and motor deficits may suggest that cognitive frailty is a risk factor for falls and disability (Rivan et al., 2021). Therefore, managing cognitive frailty should be a key focus in the aging population.

### Limitations

Several limitations should be noted in the present study. First, most participants with cognitive frailty were prefrail, representing an early transitional stage where neural and functional changes first emerge. Findings should therefore be interpreted as reflecting early-stage alterations; more severe cases may exhibit greater neural inefficiency and reduced reserve. Another factor that may affect the generalizability of our findings is that most participants were female (20/25 in the cognitive frailty group and 22/25 in the healthy control group). Although the prevalence of cognitive frailty is higher among women (Qiu et al., 2022), sex-related physiological and hormonal differences may influence neural efficiency, gait characteristics, and the trajectory of cognitive decline. Future studies with a more balanced sex distribution are needed to clarify potential gender-specific mechanisms. Second, the present study has a relatively small sample size, which may inflate type II error and fail to detect subtle difference. Future studies are encouraged to include larger samples to strengthen the evidence base. Third, this study is a cross-sectional study, which precludes causal inference regarding the relationship between brain activity and functional performance. Future longitudinal studies are needed to track neural and behavioral changes over time and to clarify the directionality of these associations. In addition, although fNIRS was selected for its portability and suitability for naturalistic walking conditions, its limited penetration depth restricts measurement to cortical regions. Consequently, potential subcortical contributions to cognitive-motor control could not be examined, and future work integrating fNIRS with neuroimaging modalities such as fMRI may provide a more comprehensive understanding.

## Conclusion

This study provides insight into cognitive-frailty-related functional brain deviations through hemodynamic changes during cognitive-motor tasking. Older adults with cognitive frailty demonstrated a certain degree of neural reserve to cope with cognitive-motor tasks; however, capacity limitations became evident when the task demand was sufficiently high. Additionally, neural inefficiency was observed as the difficulty level of cognitive-motor task increased. Our findings advance the understanding of neural mechanisms underlying cognitive frailty in older adults, which may contribute to early identification of this condition. Future studies are warranted to develop and evaluate interventions that modulate these neural alterations and enhance functional performance.

## Data Availability

The original contributions presented in the study are included in the article/supplementary material, further inquiries can be directed to the corresponding author.

## Footnotes

^1^ https://clinicaltrials.gov/
